# Newtype single-layer magnetic semiconductor in transition-metal dichalcogenides VX_2_ (X = S, Se and Te)

**DOI:** 10.1038/srep32625

**Published:** 2016-09-07

**Authors:** Huei-Ru Fuh, Ching-Ray Chang, Yin-Kuo Wang, Richard F. L. Evans, Roy W. Chantrell, Horng-Tay Jeng

**Affiliations:** 1 Graduate Institute of Applied Physics, National Taiwan University, Taipei 106, Taiwan; 2 Center for General Education and Department of Physics, National Taiwan Normal University, Taipei 106, Taiwan; 3 Physics Department, The University of York, York, YO10 5DD, UK; 4 Department of Physics, National Tsing Hua University, Hsinchu 30013, Taiwan; 5 Institute of Physics, Academia Sinica, Taipei 11529, Taiwan

## Abstract

We present a newtype 2-dimensional (2D) magnetic semiconductor based on transition-metal dichalcogenides VX_2_ (X = S, Se and Te) via first-principles calculations. The obtained indirect band gaps of monolayer VS_2_, VSe_2_, and VTe_2_ given from the generalized gradient approximation (GGA) are respectively 0.05, 0.22, and 0.20 eV, all with integer magnetic moments of 1.0 *μ*_*B*_. The GGA plus on-site Coulomb interaction *U* (GGA + *U*) enhances the exchange splittings and raises the energy gap up to 0.38~0.65 eV. By adopting the GW approximation, we obtain converged G0W0 gaps of 1.3, 1.2, and 0.7 eV for VS_2_, VSe_2_, and VTe_2_ monolayers, respectively. They agree very well with our calculated HSE gaps of 1.1, 1.2, and 0.6 eV, respectively. The gap sizes as well as the metal-insulator transitions are tunable by applying the in-plane strain and/or changing the number of stacking layers. The Monte Carlo simulations illustrate very high Curie-temperatures of 292, 472, and 553 K for VS_2_, VSe_2_, and VTe_2_ monolayers, respectively. They are nearly or well beyond the room temperature. Combining the semiconducting energy gap, the 100% spin polarized valence and conduction bands, the room temperature T_*C*_, and the in-plane magnetic anisotropy together in a single layer VX_2_, this newtype 2D magnetic semiconductor shows great potential in future spintronics.

The semiconductor-based spintronics has attracted worldwide attention in recent years because of the allowable spin current transport without the presence of a net charge current, which could avoid problems arising from capacitances and Joule heating^1^. For example, the spin pumping^2^ or the spin Seebeck effect^3^,^4^ have successfully created pure spin currents by using the thermal gradients across a ferromagnetic layer. In many cases, it involves Y_5_Fe_3_O_12_ (YIG) as the magnetic insulator and Pt as the spin current detector^5^,^6^, in which the spin current is transformed into an observable transverse voltage by the inverse spin Hall effect^7^,^8^. A new type of magnetoresistance (MR) in a Pt-YIG hybrid structure has been discovered^5^,^9^ and used in transforming magnetic data and memory storage^10^,^11^,^12^,^13^,^14^.

The next generation spintronic devices can base on room temperature ferromagnetic semiconductors or heterostructures combining ferromagnetic metals with non-magnetic semiconductors. Nevertheless searching for semiconducting materials with strong ferromagnetism and higher T_*C*_ is extremely difficult due to the conflicting requirements in the crystal and electronic structures of semiconductors and ferromagnets^12^. To date all the discovered ferromagnetic semiconductors exhibit magnetic order below room temperature, e.g., EuO (T_*C*_ = 77 K^15^), BiMnO_3_ (T_*C*_ = 100 K^16^), La_2_NiMnO_6_ (T_*C*_ = 280 K^17^), and diluted magnetic semiconductor (DMS) such as the prototypical system (Ga, Mn)As and the newly reported (Ba_1−*x*_K_*x*_)(Zn_1−*y*_Mn_*y*_)_2_As_2_ (T_*C*_ = 185 K, 180 K^18^). The only exception is the ferrimagnetic insulator Y_3_Fe_5_O_12_ (YIG) with a very high T_*C*_ = 550 K^19^ far beyond room temperature. This is the reason why most of the spintronics related works rely on YIG. Meanwhile all of the know magnetic semiconductors belong to 3-dimensional bulk materials.

Two-dimensional materials such as graphene, boron nitride, and transition metal dichalcogenides (TMDs)^20^,^21^,^22^ with the single-layer thickness less than 1 nm have attracted tremendous attention in recent years. Because of the more than 40 different families^23^,^24^,^25^,^26^ and the rich electronic properties that can create extensive applications, the TMD has become a rapidly growing research field in the past few years. Representative TMDs such as MoS_2_, MoSe_2_, WS_2_, and WSe_2_ in the monolayer (ML) form are identified as direct-band-gap semiconductors. With the time reversal symmetry preserved, giant spin splittings of 148–456 meV resulting from missing inversion symmetry and existing spin-orbit coupling^20^,^27^,^28^ could be of high potential in spintronics. However, it is still a challenge to coordinate the TMDs into nanoelectronics^29^,^30^. Therefore, developing a new type 2D TMD with exotic electronic properties is imperative.

Very recently, a new 2D TMD, few layer Vanadium disulfide (VS_2_), has been synthesized experimentally^31^,^32^. The intrinsic ferromagnetism and potential applications attract particular interests^31^,^33^,^34^. The magnetic moments and magnetic coupling strength of the ultrathin VS_2_ nanosheets could be tuned by the isotropic strain^34^. The ferromagnetism provides a new opportunity to fabricate ferromagnetic 2D TMDs without introducing magnetic transition metal atoms or tensile strains^34^. The synthesis procedures are flexible for other VX_2_ such as VSe_2_ and VTe_2_ monolayers. Besides the bulk VX_2_^25^, recent phonon dispersion calculations also reveal that monolayer VX_2_ are stable^24^.

In this work, we systematically investigate the electronic structures of monolayer and multilayer VX_2_ (X = S, Se and Te) in the 2H-phase based on the generalized gradient approximation (GGA) within the density function theory (DFT). We demonstrate that H-VX_2_ monolayers exhibit indirect semiconducting energy gaps with intrinsic ferromagnetism and in-plane magnetic anisotropy, achieving an exceptional 2-D magnetic semiconductor group. The magnitude of the band gap and even the metal-insulator transition (MIT) are tunable by applying the in-plane strain and/or changing the number of stacking layers. The GGA plus on-site Coulomb interaction (GGA + *U*) scheme, the GW approximation, as well as the HSE functional have been adopted to account for the strong correlation effect in transition-metal ions and for the well-known underestimation of the semiconducting band gaps. The Curie temperatures (T_*C*_) estimated by Monte Carlo simulations are close to or well beyond the room temperature, which makes this TMD family of high potential in real applications.

## Computational Details

The electronic structure calculations of bulk and monolayer VX_2_ are performed using the projector augmented wave (PAW) method with the Perdew-Burke-Ernzerhof (PBE) generalized gradient approximation (GGA)^35^ as implemented in the VASP package^36^,^37^. The energy cutoff of 400 eV is used for the plane-wave basis expansion with the total energy convergence criteria of 1 × 10^−5^ eV per unit cell. Gamma-centered k-grids 16 × 16 × 1 were sampled over the 2D Brillouin Zone. Optimized monolayer structures are obtained with the residual force and stress less than 0.01 eV/Å and 1.0 kBar, respectively. For few layered 2H-VX_2_ calculations, the van der Waals corrections (vdW-DF)^38^ are adopted to optimized the lattice structural parameters and bondlengths. With vdW correction, the intra-layer bond lengths are hardly changed, while the inter-layer bondlengths are significantly reduced, confirming that the interactions between VX_2_ layers of weak and non-local vdW type force. The on-site Coulomb energy *U* = 2 eV and *J* = 0.87 eV for V 3d electrons^39^ are taken into account for the electron-electron correlation effects of the localized V-3*d* orbitals in the GGA + *U*^40^ calculations. To correct the underestimated semiconducting band gaps, the GW formalism within a many-body quasiparticle framework is utilized to amend errors in the one-electron Kohn-Sham eigenvalues^41^,^42^. The Green’s function G_0_ and the screened potential W_0_ in the GW approach (G_0_W_0_) based on GGA and GGA + *U* ground states^43^ is adopted and henceforth denoted by GW for simplicity. The number of conduction bands n_*c*,max_ = 591 is sufficient to clearly resolve the peak structure of the imaginary part of the dielectric function. Similar to refs 44 and 45, the convergences of G0W0 energy gaps of VX_2_ monolayers upon the k-point mesh and the vacuum thickness have been carefully examined with the k-point mesh ranging from 12 × 12 × 1 to 30 × 30 × 1 and the vacuum thickness ranging from 15 Å up to 70 Å and then extrapolated to the infinite vacuum thickness limit. To go beyond the standard GGA approach, calculations based on the HSE^46^ functional have also been performed for comparison with the converged G0W0 energy gaps.

## Results and Discussion

### VX_2_ monolayer structure

**Figure 1:**
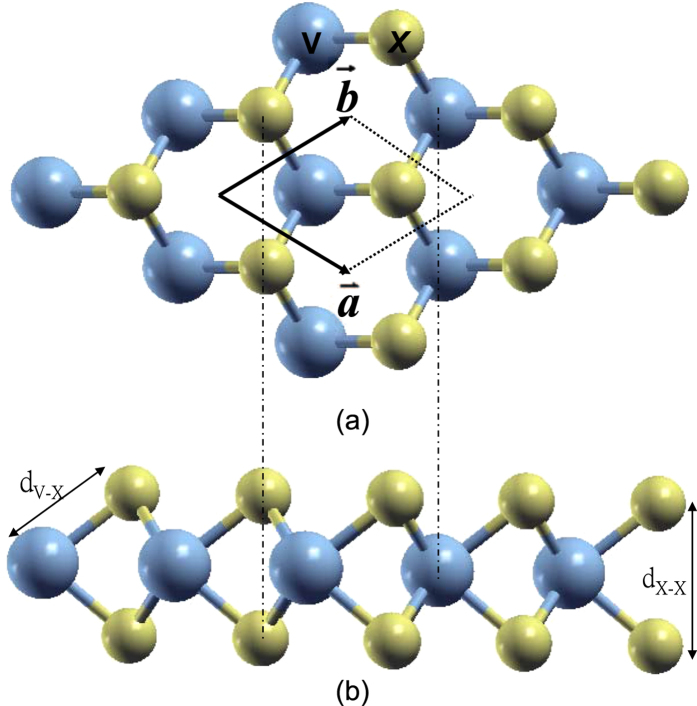
The 1H lattice structure of the monolayer VX_2_ (X = S, Se, and Te). (**a,b**) are the top and side views, respectively. 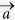 and 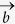 are the primitive lattice vectors of the 2D hexagonal unit cell.

The bulk VX_2_ (X = S, Se, and Te) can be formed in two common TMD structures: 2H (D_3*h*_) and 1T (D_3*d*_) polymorphs^25^,^31^,^33^. The 2H-TMDs contain two hexagonal monolayers in the unit cell with an AbA stacking sequence in each layer that the V ion is sandwiched by two X ions as shown in Fig. 1. The 1T unit cell consists of only one trigonal monolayer with an AbC stacking sequence in the monolayer. The bulk and multilayer VS_2_ in the 2H and 1T structures have been synthesized in recently years^33^,^47^,^48^,^49^,^50^. However, the bulk VSe_2_ and VTe_2_ and multilayer VSe_2_ can only be synthesized in the 1T structure to date^51^,^52^,^53^,^54^. The 1T-VX_2_ (X = S, Se, and Te) monolayer series is metallic with higher formation energies than the 2H-VX_2_^33^, therefore they are not considered in this work.

Figure 1 shows the 1H lattice structure of the monolayer VX_2_. Each V ion is surrounded by six neighboring X ions, while each X ion has three neighboring V ions. The V ions occupy the center of a trigonal prism spanned by the X ions (point group P
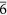
M2 (D_3*h*_)). The structural parameters of monolayer VX_2_ are determined by the geometry optimization as shown in Table 1. The calculated lattice constant of VS_2_ and VSe_2_ are 3.173 and 3.325 Å, respectively, being in excellent agreement with previously calculated results^34^. The V-X bondlength, the interlayer distance between the two X layers and between the V and X layers also agree well with previous studies^34^. The geometrically optimized VTe_2_ monolayer has the largest structural parameters because of the larger atomic radius among the three materials.

### Ferromagnetism and magnetic anisotropy

**Figure 2:**
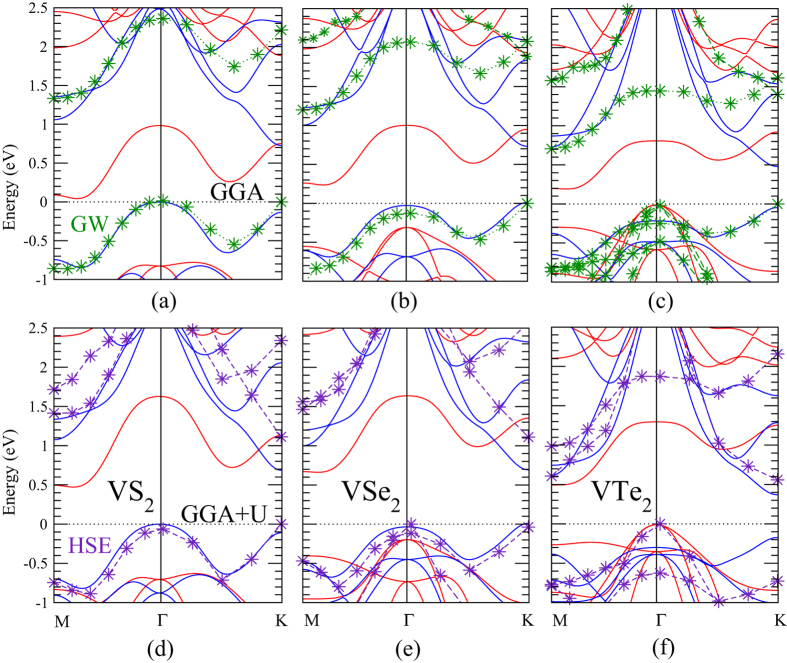
Spin-polarized band structures of VX_2_ monolayers without (upper panels) and with (lower panels) the on-site Coulomb energy (GGA + *U*) *U* = 2.0 eV and *J* = 0.87 eV for V 3d orbitals. The blue and red lines denote the spin up and down channels, respectively. The E_*F*_ (dotted horizontal line) is set at 0 eV. The GW/HSE highest valence and lowest conduction bands are denoted by green/purple star symbols in the upper/lower panels.

The spin-polarized band structures of monolayer VX_2_ along the high symmetry lines are shown in Fig. 2. The upper and lower panels show GGA and GGA + *U* results, respectively. The GGA calculations show spin polarized bands around the Fermi level (E_*F*_) with indirect band gaps of 0.05~0.22 eV originated from the exchange splitting of the V-*dz*^2^ bands, and integer magnetic moments of 1.0 *μ*_*B*_/f.u. for all the three cases. These results lead to the desirable 2D ferromagnetic semiconducting ground state. To examine the possible antiferromagnetism (AFM), we have adopted the 2 × 2 × 1 supercell for the stripe type AFM arrangement. The calculated total energies demonstrate the ferromagnetic (FM) ground state for all the three VX_2_ monolayers, being consistent with previous studies^34^,^55^,^56^.

The Stoner criterion has been successfully applied to predict the magnetism of different compounds^57^. If the Stoner criterion^58^ *ID*(*E*_*F*_) ≥ 1 is satisfied, then the compound is ferromagnetic in nature at T = 0, where D(E_*F*_) is the density of states (DOS) at E_*F*_ and I is the Stoner parameter measuring the strength of the exchange correlation. Here we adopt this criterion to examine the observed magnetism of VX_2_. Given from previous first principles calculations, the Stoner parameter I for the V atom is 0.8^59^. Non-magnetic calculations show large D(E_*F*_) values of 3.7, 4.4, 5.4 states per eV per atom for VS_2_, VSe_2_, and VTe_2_, respectively. The high DOS at the Fermi level thus cause the Stoner instability, leading to the exchange splits and the intrinsic 2D ferromagnetic ordering.

**Figure 3:**
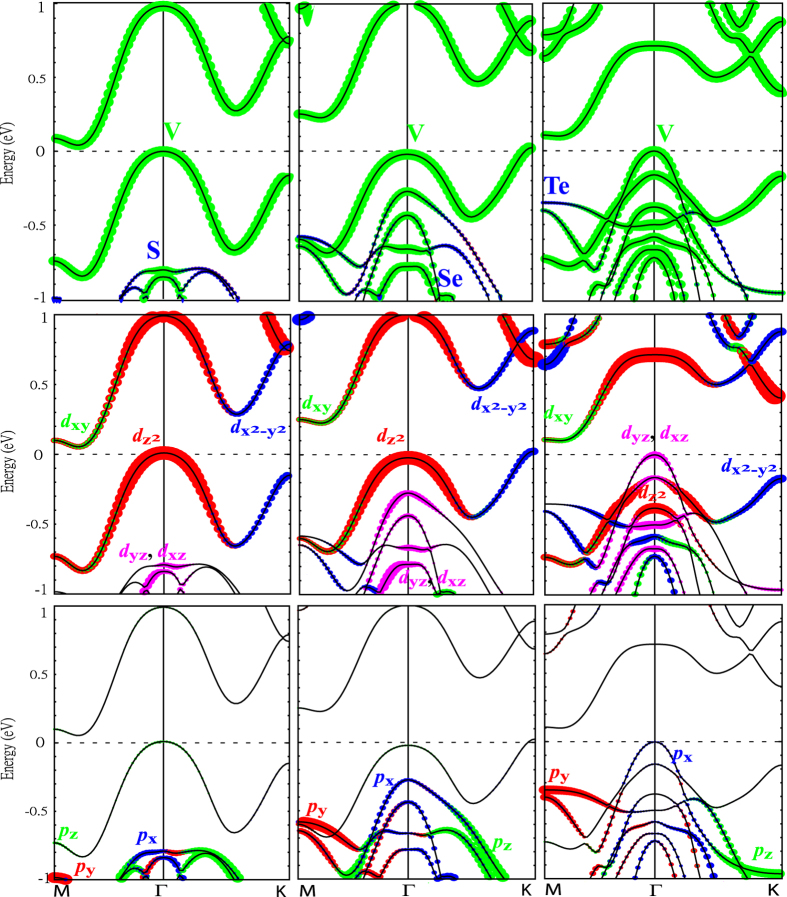
Atom and orbital decomposed band structures of VS_2_ (left hand side panels), VSe_2_ (middle panels), and VTe_2_ (right hand side panels) from GGA with spin-orbit coupling (SOC) included self-consistently. The upper panels are the atom-decomposed band structures with the green and blue colors denote the components from V and X ions, respectively. The middle panels and bottom panels are the orbital decomposed band structures for V and X ions, respectively. The colors represent the contributions from different orbitals as indicated in the figures.

The atom and orbital decomposed band structures of VX_2_ monolayers from GGA calculations with the spin-orbit coupling (SOC) included self-consistently are shown in Fig. 3. Due to the weak SOC in 3d element V, the overall band structures are more or less the same as the non-spin-orbit counterparts shown in Fig. 2. The atom-decomposed band structures in the upper panels show that the V ion predominates the top most spin up valence band and the lowest spin down conduction band around the E_*F*_, while the X ion contributes only slightly to these bands, indicating the 2D ferromagnetism originates mainly from the V ions rather than from the X ions. The *d*-orbital-decomposed band structures of V ions are depicted in the middle panels of Fig. 3. For VS_2_ monolayer, the V-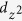 orbital predominates the valence band maximum (VBM) at Γ and the conduction band minimum (CBM) around M are mainly the 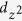 and *d*_*xy*_ hybridized states. While for VSe_2_, the V-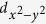 electrons dominate the VBM at K and the CBM around M are mainly the 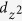 and *d*_*xy*_ mixed states. As for the VTe_2_ case, the VBM at Γ is replaced by the V-*d*_*yz*_ and *d*_*xz*_ hybridized bands arising from lower energies with the CBM around M being the 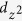 and *d*_*xy*_ hybridized states. The bottom panels of Fig. 3 illustrate the orbital contributions from the X ions. All the S, Se and Te ions play insignificant roles in the bands near the Fermi level. The above results demonstrate that these ~100% spin polarized bands around E_*F*_ in VX_2_ monolayer stem mainly from the the V-3d local moments, being consistent with previous reports^32^. This new 2D ferromagnetic semiconducting material not only provides controllable spin current applications but also regards the spin filter devices by tuning the Fermi level appropriately. Note that the band structures of VX_2_ are unlike the MoS_2_ ones. In the latter case, giant spin splittings of 148–456 meV, resulting from the stronger spin-orbit coupling in 4d orbitals and the missing inversion symmetry^26^, can be found around the K-points with spin bands degenerate elsewhere, reserving the global time reversal symmetry in MoS_2_.

Table 2 shows the calculated magnetocrystalline anisotropy energies (MAE) of the VX_2_ monolayers in the optimized structures. MAE, the total energy difference between two magnetization directions, serves as a measure of the magnetic easy-axis of a material. The total energy calculations are performed with the spin-orbit coupling included self-consistently over the 20 × 20 × 1 k-mesh in the 2D Brillouin Zone under the energy convergence criteria of 10^−8^ eV. The out-of-plane MAE is the energy difference between the perpendicular [001] and parallel [100] magnetization directions defined as E[100]-E[001]. While the in-plane MAE, defined as E[100]-E[010], is the energy difference between two in-plane magnetization directions [100] and [010]. The out-of-plane MAE of VS_2_, VSe_2_, and VTe_2_ are −0.21, −0.60, and −1.78 meV/f.u., respectively. These negative out-of-plane MAEs indicate the in-plane magnetic anisotropy (in-plane easy axis of magnetization) for all the three VX_2_ monolayers, yielding the BKT magnetic transition in the 2D XY model for future spintronic applications. As for the in-plane MAEs, the calculated E[100]-E[010] values are 2 order of magnitude smaller and play insignificant roles only.

### Band gap properties

The calculated energy gaps from GGA, GGA + SO, GGA + *U*, GGA + *U* + SO (*U* = 2, *J* = 0.87 eV), GW, as well as HSE for geometrically optimized VX_2_ (X = S, Se, and Te) monolayers are all listed in Table 3 for a systematical comparison. In the GGA scheme, the calculated indirect band gaps are 0.046, 0.225, and 0.201 eV for VS_2_, VSe_2_, and VTe_2_ MLs, respectively. As shown in Fig. 2, the CBM of VS_2_ is close to the M-point along the Γ-M direction and the VBM is located at the Γ-point. The overall band dispersion of VSe_2_ is similar to the VS_2_ one. The only difference is that the VBM goes to the K-point. The band structures of VTe_2_ are different from the previous ones in two aspects: Firstly, the VBM moves back to the Γ-point due to the rising *d*_*xz*_-*d*_*yz*_ hybridized band from lower energies (Fig. 3). Secondly, this emergent top valence band is of the same spin as the bottom conduction band, resulting in the band gap between the same spin polarized bands (Fig. 2). This is very different from the energy gaps between opposite spin channels in VS_2_ and VSe_2_ (Fig. 2).

To examine the importance of the spin-orbit interaction on the exchange splitting gaps, we have done calculations with the spin-orbit coupling (SOC) included self-consistently. As expected for 3d transition-metal ions that the weak SOC plays insignificant roles, the overall electronic and magnetic properties remain more or less the same. The only noticeable change is in the VTe_2_ band structures (Fig. 3), in which the degeneracy of the highest valence bands at Γ is lifted and the band energy at K is lowered by the SOC, as compared with the non-spin-orbit counterparts in Fig. 2. The enhanced SOC effect in these V-3d bands is induced by Te with stronger SOC in its 5p orbitals. Hence the energy gaps of VS_2_, VSe_2_, and VTe_2_ change slightly to 0.044, 0.251, and 0.149 eV, respectively, as shown in Table 3.

To take into consideration the strong electron correlations in the relatively localized 3d orbitals, we perform GGA + *U* band structure calculations as shown in the lower panels of Fig. 2. The on-site Coulomb repulsion *U* of 2 eV enhances the exchange splitting and gives rise to larger energy gaps of 0.473, 0.651, and 0.379 eV for VS_2_, VSe_2_, and VTe_2_, respectively, as listed in Table 3. Besides the significantly raised exchange gaps, the CBM and VBM locations of VS_2_ and VSe_2_ remain the same as those from GGA. However for the VTe_2_ case, the CBM from GGA + *U* locates at the K-point rather than around the M-point given by GGA. Finally the combined SOC and on-site Coulomb repulsion *U* effect slightly changes the GGA + *U* energy gaps as listed in Table 3. The broadest energy gap obtained from standard DFT (GGA and GGA + *U*) calculations is 0.684 eV of VSe_2_, which is adequately large for real applications.

As mentioned above, the on-site Coulomb repulsion *U* of 2 eV used in this work is given from a previous theoretical estimation for V atoms^39^. Since the *U* value of the same element in different materials also depends on the ionicity and the composition of the embedded compound, the precise value of *U* in VX_2_ is actually unknown. Because of the uncertainty of the *U* value, we therefore consider the GW correction which would give the most reliable band gaps in semiconducting materials. To compare with the standard GGA band structures, the GW approximation corrected band energies with carefully examined convergence upon vacuum thickness and number of k-points are denoted by green star symbols in the upper panels of Fig. 2. For simplicity we only depict the highest valence states and lowest conduction states with the top most GW valence band energies aligned at Ef. The GW corrected VBM and CBM of VS_2_ are located at Γ- and M-point, respectively. While for VSe_2_ and VTe_2_, the VBM and CBM are located at K- and M-point, respectively. The GW corrected band gaps are raised to 1.334, 1.200, and 0.705 eV for VS_2_, VSe_2_, and VTe_2_ monolayers, respectively, as shown in Table 3. To go beyond the standard GGA and GGA + *U* approach, we also adopt the HSE functional to calculate the band structures of VX_2_ monolayers as denoted by purple star symbols in the lower panels of Fig. 2 (only bands closest to Ef are depicted). The resultant HSE energy gaps of 1.110 eV, 1.150 eV, and 0.560 eV for VS_2_, VSe_2_, and VTe_2_ monolayers, respectively, are also listed in Table 3. As can be seen, the HSE energy gaps agree very well with the converged G0W0 results at the infinite vacuum thickness limit.

**Figure 4:**
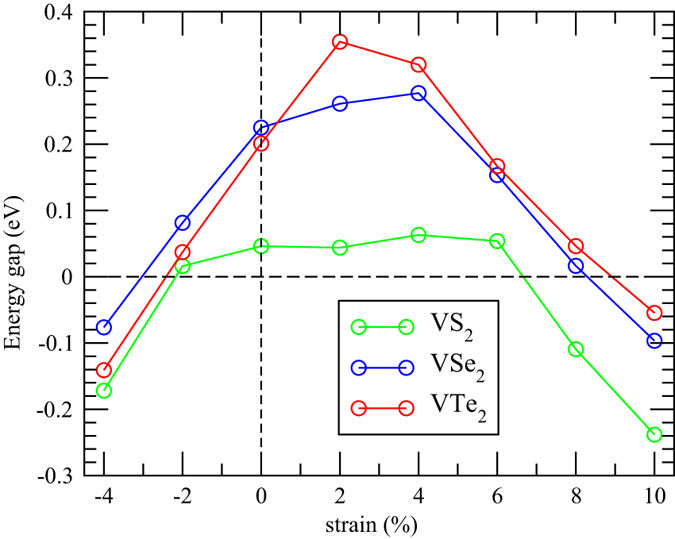
The GGA energy gap of the VX_2_ monolayer as a function of the strain.

The GGA energy gap of VX_2_ monolayer as a function of the strain from −4% up to 10% is shown in Fig. 4. For VS_2_, the energy gap remains more or less the same below 6% strain, and then turns into the gapless metallic state. While for the other two cases, the energy gap increases below 4% and then decreases to a negative value (corresponding to the band overlap value) above 8% strain. In all cases the tensile strain affects the gap size significantly and eventually induces the metal-insulator transition (MIT) as shown in Fig. 4. This is because that the unoccupied *dz*^2^ band at the K-point shifts downward below the E_*F*_ at high tensile strains due to the reduced Coulomb repulsion, and finally closes the energy gaps of the VX_2_ monolayers. We note that VTe_2_(VSe_2_) reaches the maximum energy gap of 0.36(0.28) eV under 2(4)% tensile strain. This strain-induced exchange gap enhancement implies a higher Curie temperature than the strain free one. On the other hand, due to the increased Coulomb repulsions and enhanced band dispersions of these *dz*^2^ bands under compressive strains, the energy gaps are therefore suppressed and achieve another MIT under negative strains around −2%, as can be seen in Fig. 4.

**Figure 5:**
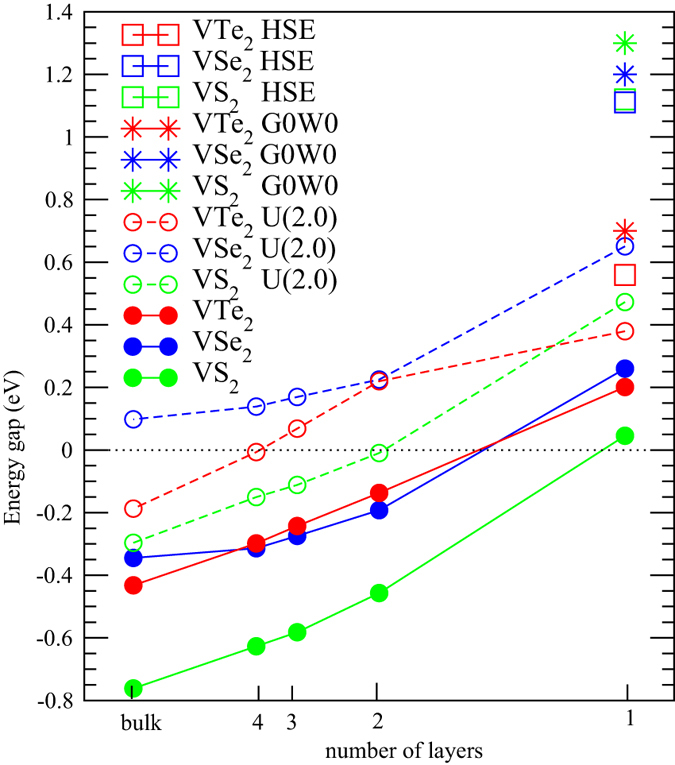
The energy gap of VX_2_ from GGA, GGA + *U*, GW, and HSE as a function of the number of layers.

So far we have demonstrated various effects on the energy gaps of the VX_2_ monolayers. Below we discuss the dependence of the energy gaps on the thickness of VX_2_ multilayers. The gap values given from GGA, GGA + *U*, GW, as well as HSE calculations are summarized in Fig. 5. First of all we would like to emphasize that all kinds of calculations performed in this work demonstrate an exchange splitting energy gap for all the three VX_2_ monolayers, strongly supporting that the VX_2_ families in the monolayer form are indeed 2D ferromagnetic semiconductors. While increasing the number of layers would enhance the interlayer interactions and band dispersions, and hence significantly suppress the energy gap. For the bilayer systems, all the GGA gaps are closed while the GGA + *U* still gives gaps of ~0.23 eV for VSe_2_ and VTe_2_. As for the trilayer and thicker systems, the GGA + *U* gap size decreases further. At the bulk limit, only VSe_2_ exhibits a gap of about 0.1 eV from GGA + *U*, while no energy gap can be found for VS_2_ and VTe_2_. In comparison with the consistent semiconducting ground state for VX_2_ monolayers and the diverse results for bilayer and thicker layers, one may conclude that the ferromagnetic semiconducting phase exists only in the monolayer VX_2_ systems. Any thicker VX_2_ multilayers would cause the instability of the ferromagnetic semiconducting phase and result in the metallic phase, giving rise to the metal-insulator transition (MIT) upon layer thicknesses. Note that the experimental synthesis of monolayer VX_2_ is not yet reported, while multilayer VX_2_ has been demonstrated as a ferromagnetic metal experimentally^32^.

### Exchange interaction parameters and Curie temperature

**Figure 6:**
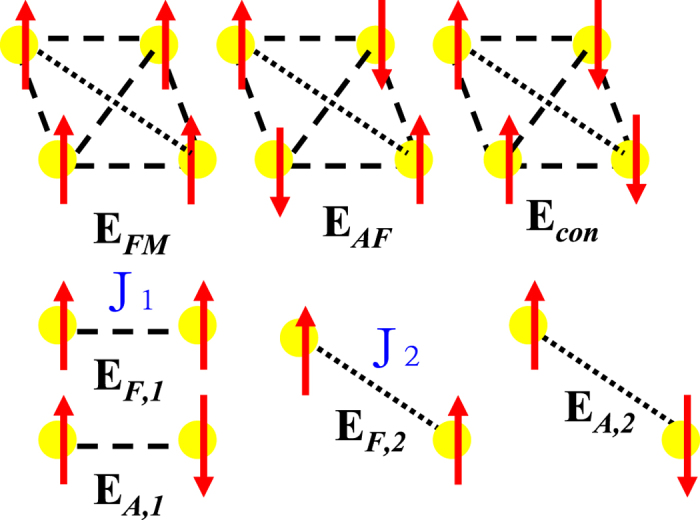
Upper panels: Schematic view of the three magnetic configurations: the ferromagnetic *E*_*FM*_, antiferromagnetic *E*_*AF*_, and collinear antiferromagnetic *E*_*col*_ spin arrangements. Lower panels: Schematic plot of the exchange parameters J_1_ and J_2_, magnetic bond energies *E*_*F*1_ and *E*_*A*1_ between the nearest V-V moments, and *E*_*F*2_ and *E*_*A*2_ between the next-nearest V-V moments.

To find the magnetic ground state of the VX_2_ monolayers, we considered three possible magnetic configurations within the 2 × 2 × 1 supercell including the ferromagnetic (FM), antiferromagnetic (AFM), and collinear antiferromagnetic (COL) spin arrangements as depicted in the upper panels of Fig. 6. The corresponding total energies of the three cases provide the estimation of the exchange interaction parameters between the nearest-neighbor (NN) couplings *J*_1_ and the next-NN (NNN) couplings *J*_2_^60^,^61^ as illustrated in the lower panels of Fig. 6. To evaluate the exchange coupling, we consider the calculated total energy of the VX_2_ monolayer based on GGA-PBE functional as the sum of the NN spin-spin interactions in terms of the spin Heisenberg model,


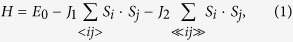


where <*ij*> and ≪*ij*≫ are respectively the summation over the NN and NNN V site i and site j, and *S*_*i*_ (*S*_*j*_) is the unit vector representing the direction of the local magnetic moment at site i(j). J > 0 is assumed for the FM interaction, and J < 0 is assumed for the AFM interaction. The constant E_0_ contains all spin-independent interactions.

To determine the values of J_1_ and J_2_, one needs to evaluate the energy difference between a pair of nearest V-V moments in parallel (*E*_*F*1_) and antiparallel (*E*_*A*1_) alignments,


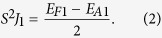


The *E*_*F*1_ is not necessary to be equal to −*E*_*A*1_ since the energy of the reference state may not be located exactly at the middle of the energy between the *E*_*F*1_ and *E*_*A*1_. Meanwhile *S*^2^*J*_2_ also evaluates the energy difference between a pair of the next-nearest V-V moments in parallel (*E*_*F*2_) and antiparallel (*E*_*A*2_) arrangements,


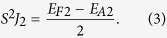


The total energies of the ferromagnetic (*E*_*FM*_), antiferromagnetic (*E*_*AF*_), and collinear antiferromagnetic (*E*_*col*_) states with respect to the nonmagnetic state (*E*_*NM*_) can be expressed by the following equations:


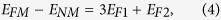



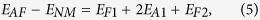



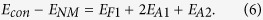


By solving the above equations with calculated total energies of the related spin states, we obtain the exchange interaction parameters J_1_ and J_2_ for VX_2_ monolayers as listed in Table 4. As shown the ferromagnetic interactions between two NN V spins are very strong, especially in VSe_2_ and VTe_2_. The exchange coupling parameter J_1_ = 38.8(44.3) meV of VSe_2_(VTe_2_) is about twice larger compared with 23.8 meV of VS_2_. On the other hand, the negative J_2_ values of VX_2_ show the antiferromagnetic coupling between two next-nearest-neighboring V spins with the values being 2~3 order of magnitude smaller than the NN couplings. Thus summation over all the J_1_ and J_2_ would give rise to the ferromagnetic ground state in the 2D VX_2_ monolayers.

With the exchange interaction parameter J available, the Curie temperature (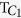) can then be estimated by J/k_*B*_ as listed in Table 4. The ferromagnetic exchange coupling of VS_2_ monolayer gives rise to the 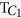 of 138 K. This is reasonably close to the experimental observations that the ultrathin VS_2_ nanosheets with the averaged thickness of ~8 nm shows clear low-temperature ferromagnetism with *T*_*C*_ = 72 K, while the Tc is decreased to 10 K for VS_2_ nanoflowers with the average thickness of ~150 nm^62^. Our calculations also show the suppressed ferromagnetic couplings and decreasing T_*C*_ upon increasing the number of layers. The calculated T_*C*_ of the bilayer VS_2_ is 124 K which is less than the 138 K of VS_2_ monolayer. More importantly, the T_*C*_ of VSe_2_ and VTe_2_ monolayers are respectively 223 K and 225 K, which are much closer to the room temperature. It is noted previously that the exchange gaps of VSe_2_ and VTe_2_ can be enhanced by tensile strains, which implies stronger exchange interaction parameters and hence higher Curie temperatures than the strain free ones. As a result, the Tc could be even closer to the room temperature by appropriate manipulations.

For comparison, we also estimate the Curie temperature (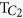) via a simplified method^63^, 
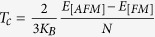
, where N is the number of magnetic ions in the unit cell. The Curie temperature 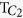 estimated from the calculated total energy differences between the AFM and FM phases of VX_2_ monolayers are listed in Table 4. As shown the T_*C*_ values of VX_2_ monolayers obtained in this way are much higher than the previous ones: all the estimated 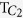 are above the room temperature. Particularly the Curie temperatures of VSe_2_ and VTe_2_ monolayers could be over 600 K.

**Figure 7:**
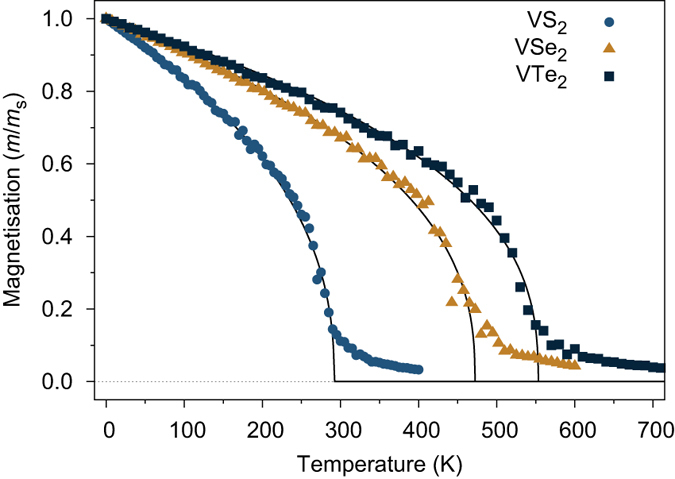
Monte Carlo simulation for the magnetization as functions of the temperature.

To accurately calculate the Curie temperature beyond the simple mean field estimations (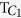 and 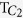), we have adopted the Monte Carlo simulations for the magnetizations as functions of the temperature. With the calculated exchange parameters J_1_ and J_2_, we simulate the Curie temperature of the monolayer system based on the Monte Carlo metropolis simulations using the VAMPIRE software package^64^,^65^. The simulated system for all materials consists of a platelet with 11172 spins with a hexagonal crystal structure. The spins are initialized along the [100] crystal direction and thermalized for 10000 equilibrium steps followed by 50000 averaging steps to calculate the thermal equilibrium magnetization at each temperature. The Monte Carlo simulations use the Hinzke-Nowak combinational algorithm^66^ for fast relaxation to thermal equilibrium. The simulated temperature dependent magnetization for VSe_2_, VS_2_ and VTe_2_ are shown in Fig. 7.

The temperature dependent magnetization is fitted using the Curie-Bloch equation in the classical limit^67^


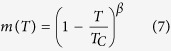


where T is the temperature, T_*C*_ is the Curie temperature and *β* ~ 0.36 is the critical exponent. The fitted Curie temperatures T_*C*_ are summarized in Table 4. The Monte Carlo simulations predict T_*C*_ values of 291 K, 472 K, and 553 K for VSe_2_, VS_2_ and VTe_2_ monolayer, respectively. All of them are close to or higher than the room temperature, which demonstrates excellent evidence for room temperature 2D magnetic semiconductors of VX_2_ monolayers. In comparison with previous estimations on the T_*C*_ values from the standard mean field expression, the Monte Carlo results are in general slightly higher than the 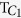
68 while lower then 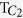
69,70 estimated from the mean field expressions.

## Conclusions

We present theoretical investigations on a new type 2D ferromagnetic semiconductor: VX_2_ (X = S, Se and Te) monolayer based on GGA, GGA + *U*, GW, as well as HSE calculations. The standard GGA scheme gives indirect exchange energy gaps of 0.046, 0.225, and 0.201 eV for VS_2_, VSe_2_, and VTe_2_ monolayers, respectively, with integer magnetic moment of 1 *μ*_*B*_/f.u. for all the three cases. The 100% spin polarized bands around E_*F*_ are mainly from the 3*d* local moments in the V ions. The weak spin-orbit interaction in V 3d electrons plays insignificant roles in the energy gaps. The MAE calculations show that the easy axes are parallel to the layers for all the three cases. The on-site Coulomb interaction *U* = 2 eV enhances the energy gaps by about 0.4 eV. The GW approximation corrected band gaps are 1.3, 1.2, and 0.7 eV for VS_2_, VSe_2_, and VTe_2_ monolayers, respectively. They agree very well with the HSE energy gaps of 1.1, 1.2, and 0.6 eV, respectively. The gap size and even the metal-insulator transitions are tunable via controlling the ambient parameters such as changing the number of layers and/or applying the strain. The theoretical evaluation on the exchange coupling constants reveals the dominant ferromagnetic coupling. Moreover our Monte Carlo simulations illustrate very high Curie temperatures of 292, 472, and 553 K for VS_2_, VSe_2_, and VTe_2_ monolayers, respectively. They are nearly or well beyond the room temperature. Our study demonstrates the great potential of the VX_2_ monolayers in spintronics and invites further experimental investigations on these ultrathin newtype room temperature 2D ferromagnetic semiconductors.

## Additional Information

**How to cite this article**: Fuh, H.-R. *et al*. Newtype single-layer magnetic semiconductor in transition-metaldichalcogenides VX_2_ (X= S, Se and Te). *Sci. Rep*. **6**, 32625; doi: 10.1038/srep32625 (2016).

## Figures and Tables

**Table 1:** Optimized lattice constant a, V-X bondlength *d*_*V*−*X*_, interlayer distance between two X layers *d*_*X*−*X*_ and between V and X layers Δ_*V*−*X*_ of monolayer VX_2_ (X = S, Se, and Te).

(Å)	a	a(Theo.)	*d*_*V*−*X*_	*d*_*X*−*X*_	Δ_*V*−*X*_
VS_2_	3.173	3.174^34^	2.362	2.982	1.491
VSe_2_	3.325	3.331^34^	2.501	3.205	1.602
VTe_2_	3.587	–	2.715	3.510	1.755

**Table 2:** Calculated magnetic anisotropy energies of the VX_2_ monolayers.

	VS_2_	VSe_2_	VTe_2_
E[100]-E[001] (meV)	−0.21	−0.60	−1.78
E[100]-E[010] (*μ*eV)	−3	−7	−8

**Table 3:** Calculated energy gaps from GGA, GGA + SO, GGA + *U*, GGA + *U* + SO with *U* = 2, *J* = 0.87 eV, GW, and HSE for geometrically optimized VX_2_ (X = S, Se, and Te) monolayers.

(eV)	GGA	GGA + SO	GGA + *U*	GGA + *U* + SO	GW	HSE
VS_2_	0.046	0.044	0.473	0.473	1.334	1.110
VSe_2_	0.225	0.251	0.651	0.684	1.200	1.150
VTe_2_	0.201	0.149	0.379	0.282	0.705	0.560

**Table 4:** Calculated exchange interaction parameters J_1_ and J_2_ and the Curie temperatures of VS_2_, VSe_2_, and VTe_2_ monolayers.

	VS_2_	VSe_2_	VTe_2_
J_1_ (meV)	23.8	38.8	44.3
J_2_ (meV)	−0.05	−0.002	−0.001
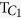 (K)	138	223	225
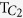 (K)	369	600	686
*β*	0.423	0.393	0.374
T_*C*_ (K)	292	472	553

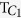
 and 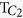 are estimated from the standard mean field expressions. T_*C*_ is calculated from the Monte Carlo simulations with *β* the critical exponent.
